# Somatotopic organization of ferret thalamus

**DOI:** 10.3389/fnint.2014.00090

**Published:** 2014-11-21

**Authors:** Mario Vázquez-García, Marie-Josée Wallman, Igor Timofeev

**Affiliations:** ^1^Le Centre de Recherche de L’Institut Universitaire en Santé Mentale de QuébecQuébec, QC, Canada; ^2^Departamento de Fisiología, Facultad de Medicina, Universidad Nacional Autónoma de MéxicoMéxico, D.F., México; ^3^Department of Psychiatry and Neuroscience, Université LavalQuébec, Canada

**Keywords:** thalamus, VPM, VPL, ferret, tactile stimulus

## Abstract

The stereotaxic reference marks of ferret skull have large variability and the reference point for stereotaxic experiments in ferret brain is difficult to define. Here, using extracellular single-unit recordings, we studied the somatotopic organization of cutaneous receptive fields in the ventroposterior medial (VPM) and the ventral posterolateral (VPL) nuclei of the ferret thalamus. The mechanical stimulation of the skin was done through air puffs. The skull was positioned according to Horsley-Clarke coordinate system. Most of the neurons responding to face skin stimulation were located +7–+9 mm anterior, 2–3.9 mm lateral and 7–9.6 mm from cortical surface, whereas those responding to body skin stimulation were located +7–+10 mm anterior, 3.3–5.5 mm lateral and 6.7–10 mm from cortical surface. Out of 90 thalamic neurons recorded in this study, 58 responded to the body and the other neurons to the face stimulation. All neurons responded with spikes to stimulus onset, 37% of neurons responded only to stimuli onset and offset and 22% neurons fired tonically throughout stimulating epoch. The whiskers representation was located in the middle of the VPM nucleus, whereas those of the tongue, nose, bridge of the nose, supraorbital areas, upper and lower lips, and lower jaw were surrounding the whiskers representation. Within the VPL nucleus there was a clear topological correspondence from forelimb to hindlimb in the medial-to-lateral direction. Our findings indicate the whiskers representation in VPM or the forelimb-hindlimb representation in the VPL nucleus can be considered as a reliable reference in the ferret thalamus.

## Introduction

The thalamus is the main relay of sensory information coming from the periphery toward the cerebral cortex in mammalians. An essential function of the thalamus is to integrate afferent information carried by periphery sensory pathways with descending corticothalamic and brainstem modulatory systems (Jones, 2001; Nicolelis and Fanselow, 2002; Sherman, 2007, 2012; Viaene et al., 2011).

The ferret represents a greatly useful animal model to study multiple aspects of central nervous systems. The high proportion of REM sleep make them useful to study transitions between states of vigilance (Marks and Shaffery, 1996; Jha et al., 2006; Thurber et al., 2008). Cortical or thalamic slices from ferrets display a variety of sleep oscillations (von Krosigk et al., 1993; Kim et al., 1995; Sanchez-Vives and McCormick, 2000; Haider et al., 2006; Sanchez-Vives et al., 2007). Multiple studies investigated cortical representations of well-developed somatosensory system of ferrets (Leclerc et al., 1993; Rice et al., 1993; McLaughlin et al., 1998; Foxworthy and Meredith, 2011) as well as other sensory systems (Rice et al., 1993; Bizley et al., 2005; Manger et al., 2010).

Little is known about topographically identified subcortical structures in ferret brain (King and Hutchings, 1987; Duque and McCormick, 2010; Manger et al., 2010). The standard stereotaxic reference marks of the ferret skull have large variability (Lawes and Andrews, 1987), making difficult to build an atlas of ferret brain. A relative location of different thalamic nuclei of ferret has been described (Herbert, 1963) without any reference to known stereotaxic coordinate systems.

When the head is fixed to a stereotaxic frame the stereotaxic errors are usually smaller for closely located structures. Having some reference point(s) in the thalamus would reduce errors of calculation of other thalamic nuclei. Because delivery of somatosensory stimuli is easy to achieve in any electrophysiological laboratory that uses an *in vivo* recording approach, it can be promising to use specific locations in the somatosensory thalamus as reference points to identify other subcortical structures in the ferret brain, however, the location of the somatosensory thalamus and the precision of structure identification in ferrets’ brain are unknown.

The ventrobasal complex in the dorsal thalamus is composed of the ventral posterior medial (VPM) and the ventral posterior lateral (VPL) nuclei of the thalamus (Rose and Mountcastle, 1952; Andersen et al., 1964; Loe et al., 1977) with face representation in the VPM nucleus and body representation in the VPL nucleus. To the best of our knowledge, neuronal responses to body stimuli were not investigated before in the thalamus of ferrets. The goal of this study was to provide information on the somatotopic representation of the body surface within the VPM and VPL thalamic nuclei of the ferret thalamus. In this study we describe the localization of somatosensory thalamus of adult ferrets with the cranium positioned according to the Horsley–Clarke coordinate system.

## Materials and methods

The experimental data described here were obtained from nine experiments performed in adult *Mustela putorios furo* male ferrets (0.9–1.55 kg). Experiments were carried out in accordance with the guidelines published in the NIH Guide for the Care and Use of Laboratory Animals (NIH publication no. 86–23, revised 1987) and were approved by the Committee for Animal Care of Université Laval.

The animals were deeply anesthetized with isoflourane (1.75–2.25%). The EEG and the absence of withdrawal reflexes were regularly verified to ensure an adequate level of anesthesia. 3.3% dextrose in 0.3% NaCl solution was given i.v. (0.016 ml/min) to prevent hypovolemia. Heart rate (200–240 beats min^−1^), blood oxygen saturation (>95%) and rectal temperature (near 38°C) were continuously monitored and kept within normal limits. Xylocaine 2% or bupivacaine 0.5% were applied over all pressure points and over the wound edges. Ophthalmic ointment was applied onto corneal lens surface. The animals were placed on a stereotaxic frame according to Horsley-Clarke coordinates with ferret adapters from David Kopf Instruments (Tujunga, CA, USA). Under aseptic surgical conditions the skin and the muscles of the head were gently removed from the top of the skull. The EEG was recorded via a 1 mm diameter steel screw juxtaposed to the dura mater above the right parietal cortex. In order to facilitate access to the somatosensory thalamus a craniotomy with a diameter of about 2 cm was performed over the left hemisphere, and the dura mater was removed. Mineral oil was applied to prevent cortex from drying. To reduce brain pulsations arising from the heartbeat and respiration, a cisternal drainage was made and after the high impedance tungsten electrode was in place over the presumed somatosensory thalamus, a warm isotonic saline solution of 4% agar was placed over the craniotomy. At the end of experiment, animals were given an overdose of ketamine (12–15 mg/kg, i.v.) and xylazine (1 mg/kg, i.v.) and perfused transcardially with 0.9% saline followed by 4% paraformaldehyde (PFA) in 0.1 M phosphate-buffered saline (PBS) and finally by 4% sucrose in 0.1 M PBS. After fixation, the head was again placed in the stereotaxic frame. One large surgical needle (18G) was inserted vertically at the anterio-posterior plane 0 mm and laterally at 5 mm; another needle was inserted horizontally at the anterio-posterior plane +2 mm and medio-laterally at a depth of 5 mm. The brains were removed and processed by Nissl histology. The traces left by needles were used as references to align orientation of brains during sectioning.

Evoked and spontaneous extracellular single-unit neuronal activities were recorded from the ferret thalamus using epoxylite-insulated tungsten electrodes with 9–12 MΩ impedance. An additional electrode was inserted in the somatosensory cortex for continuously recording the spontaneous oscillatory activity of local field potential (LFP), which was also used to monitor the level of anesthesia. An A-M Systems High-Gain AC/DC differential amplifier was used in conjunction with Nicolet Vision 2.0 for data acquisition, and off-line analysis was performed with Igor Pro (version 4.0).

Recording electrodes were aligned to the stereotaxic frame; where the zero point of L and AP axes in Horsley-Clarke coordinates correspond to midline and intraaural position in the horizontal plane, and the zero point of the vertical (D) axis corresponds to pial surface. Each track series began medially at L 1 mm and at +12 mm in AP direction. The electrodes were lowered up to 12 mm from pial surface. In successive tracks the electrodes were moved first laterally and then posteriorly. The thalamic region was identified by the presence of typical high-frequency spike-bursts, which always appeared after a depth of 4 mm. From that point the electrodes were advanced slowly to obtain 1–4 well-isolated neuronal units (typically single units). The area with single-unit activity responsive to cutaneous stimulation was initially identified by touching the contralateral body surface. The location of the receptive field (RF) was first explored with a handheld cotton applicator, where slight touch evoked neuronal firing. Thereafter, mechanical stimuli were delivered with quantitative and timed Picospritzer pressure system for ejection of air volumes (Air puff) and triggered from an external stimulator. Data were obtained in sets of 300 air puffs of 200-ms duration and 80-PSI pressure delivered at regular intervals of 1 s. The distance between the tip of the tube, which directed the air to the responsive surface of skin, was 3 mm. The diameter of the stimulated skin area was 2–3 mm.

The topographic organization of the ferret somatic sensory thalamus was determined by a point-to-point correspondence between the recording site location and location of the respective neuronal receptive field in the cutaneous surface. The stereotaxic coordinates of the recording site and the location of the receptive field on the body were recorded for each identified single unit. Systematically, successive electrode tracks were spaced apart at a distance of 0.2 or 0.5 mm in the medial-to-lateral (L) and in the anterior-to-posterior (AP) axes within the entire thalamic region that was responsive to tactile stimulation of the body. The data groups of stereotaxic coordinates were compared using Kruskal-Wallis Test followed by Dunn’s Multiple Comparisons Test. Parameter values are expressed as mean ± standard error. Finally, the position of neurons responsive to cutaneous stimulation was presented on frontal, sagittal and horizontal histological sections.

## Results

**Figure 1:**
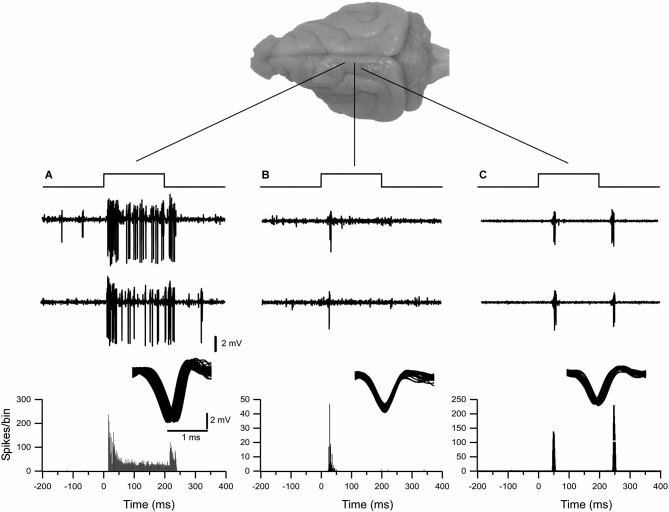
**Examples of typical extracellular single-unit responses obtained from VPM and VPL thalamic nuclei. (A)** A VPL neuron responds tonically to tactile stimulation of upper lip. **(B)** A neuron from the VPM nucleus responded with spikes on whisker stimulus ON. **(C)** A neuron from the VPL nucleus responded with spikes on both stimulus ON and stimulus OFF applied to hind paw. Three upper traces in **(A)**, **(B)** and **(C)** show typical responses to tactile stimulation of thalamic neurons. Below, the peristimulus spike histogram (bin 1 ms).

In our experimental conditions, a total of over 90 recording sites with single-unit neuronal activity were obtained from the ferret somatosensory thalamus, of which 32 correspond to the representation of the head and 58 correspond to the body. Single-unit activity recordings demonstrated robust and reliable tactile-evoked responses. The air-puff-tactile stimulation evoked three types of single neuronal activity. All thalamic neurons reported in this study fired short-latency spikes at the onset of stimulus. Thirty seven percent of neurons responded to the stimulus onset and offset and 22 % of neurons were tonically responsive throughout the 200 ms of stimulation (Figure 1).

### Functional identification of VPM nucleus

**Figure 2:**
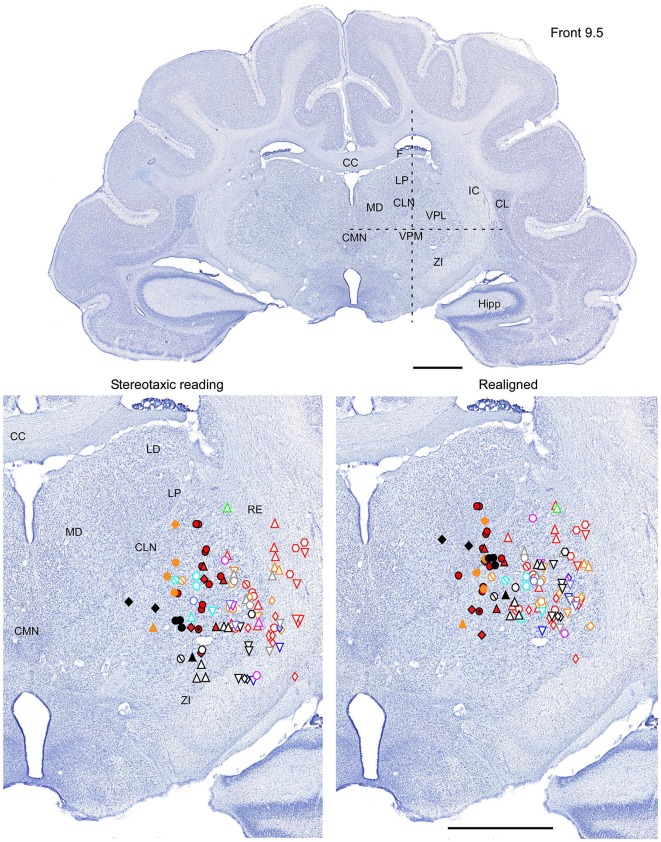
**Mapping of units receptive fields in the VPM and VPL thalamic nuclei (coronal view)**. Upper panel shows the full coronal section. Dotted lines in the upper panel show the location of horizontal section (Figure 3) and sagittal section (Figure 4). *Lower left* panel shows the location of recording sites from all experiments aligned according to stereotaxic coordinates. *Lower right* panel shows the location of recording sites from one experiment (red symbols) aligned according to stereotaxic coordinates, and other experiments realigned according to the center of the whisker receptive field of that experiment. Data from nine ferrets are shown. Filled symbols indicate recording sites of face: whiskers (●), nose region (▲), lips (⊙), and lower jaw (◆). Empty symbols indicate recording sites of (1) forelimb: digits (○), paw, wrist, leg, and elbow (△); (2) Hindlimb: digit (◊), paw, knee, and thigh (▽); and (3) tail and back (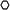) and neck (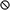). Different colors represent different experiments. Scale = 2 mm.

**Figure 3:**
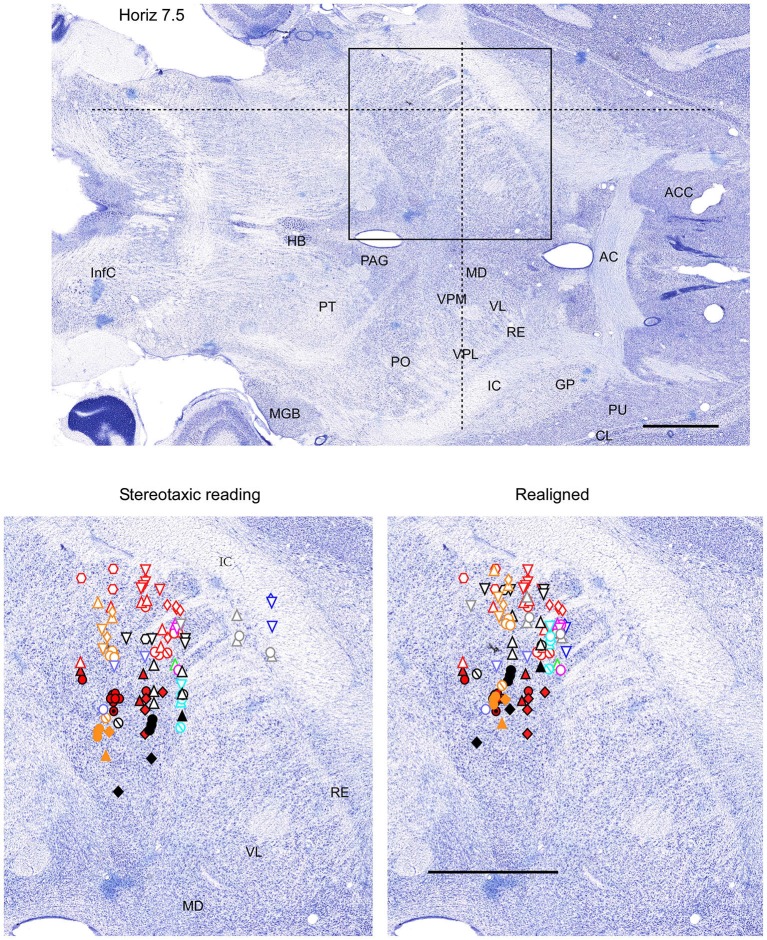
**Mapping of units receptive fields in the VPM and VPL thalamic nuclei (horizontal view)**. Data from nine ferrets are shown. All symbols are as in Figure 2. The symbols represent data from the same experiments as in Figures 2, 4. Dotted lines in the upper panel show the location of coronal section (Figure 2) and sagittal section (Figure 4). Scale = 2 mm.

**Figure 4:**
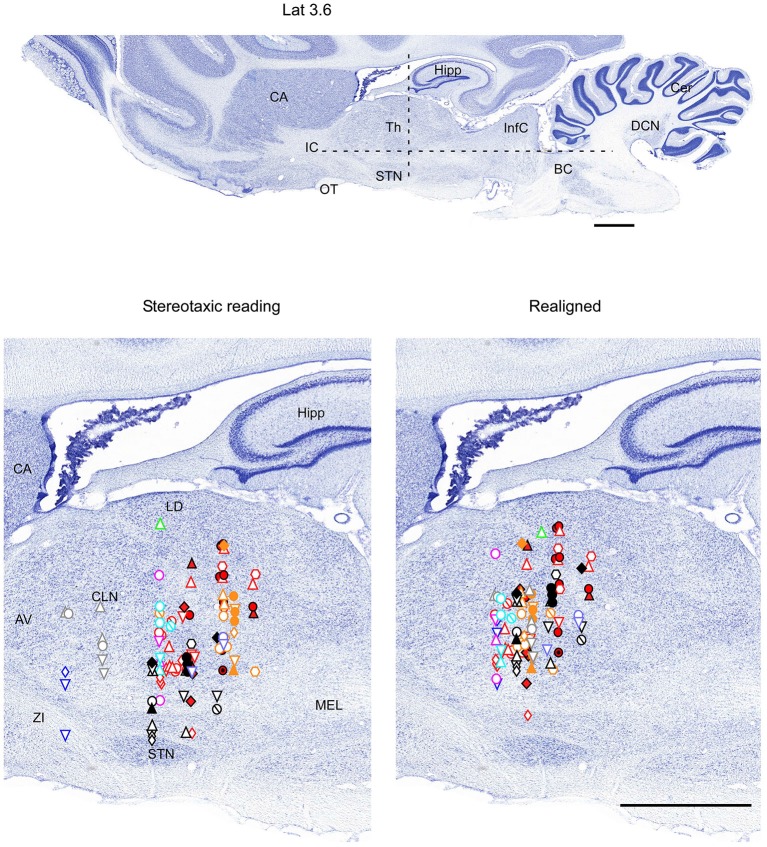
**Mapping of units receptive fields in the VPM and VPL thalamic nuclei (sagittal view)**. Data from nine ferrets are shown. All symbols are as in Figure 2. Dotted lines in the upper panel show the location of coronal section (Figure 2) and horizontal section (Figure 3). Scale = 2 mm.

All nine animals exhibited the same general somatotopic organization pattern: the face and tongue representations were medial (VPM), and the neurons responding to the stimulation of the body were located laterally (VPL). Figures 2–4 (bottom left) show the distribution of recording sites containing neurons that responded with spikes to somatosensory stimulation according to stereotaxic coordinates. Within the VPM nucleus, 51% of units had RF on the whiskers, usually involving at least two whiskers. In each experiment we found that the whiskers representation was generally located in the middle of the VPM, whereas neurons responding to the tongue, nose, bridge of the nose, supraorbital areas, upper and lower lips, and lower jaw were surrounding the whiskers representation (Figures 2, 3). In all animals, most of the neurons responding to face skin stimulation and whiskers were located +7–+9 mm anterior, 2–3.9 mm lateral and 7–9.6 mm from cortical surface. Such relatively large variability was likely due to a large variability in skull features. In one of the experiments, we recorded 44 single units (17–face, 12–foreleg, 11–backleg, 1 neck, and 3–trunk). Out of them 9 were activated by whisker stimulation. All neurons responding to whisker stimulation were located at AP between 7 mm and 8 mm (8 of them between 7.5 mm and 8 mm), lateral 3.8 mm and depth 8.3 mm, depth 7.3 mm and 8 mm. This result suggests that neurons responding to whiskers stimuli are localized in a very compact thalamic zone, which might be used as a reference point to search for other subcortical structures.

### Functional identification of the VPL nucleus

Fifty-eight single-unit neuronal responses with tactile RFs located on the contralateral body were recorded in the VPL thalamic nucleus. There was an obvious topological correspondence from forelimb to hindlimb along the medial-to-lateral axis of the VPL nucleus. Neurons with tactile RFs on the forelimb were more medially located than neurons with RFs on the hindlimb (Table 1). Our sample indicates that the largest representation in the VPL nucleus corresponds to digits of both fore paws and hind paws. A large number of single-units had RFs on the digits of the forelimb (42%), and on the digits of the hindlimb (41%). The representation of the remaining parts of the forelimb (ventral and dorsal paw, leg, elbow, etc.) and of the hind limb (ventral and dorsal paw, leg, heel, thigh, etc.) corresponded to 52% and 51% of the single-units, respectively. RFs located at tail and trunk were usually very large. These RFs were recorded from neurons in caudal and lateral locations in the VPL. Neurons responding to body skin stimulation (VPL nucleus) were located in +7–+10 mm anterior, 3.3–5.5 mm lateral, and 6.7–10 mm from cortical surface (Table 1).

**Table 1:** **Averaged values of the stereotaxic coordinates of the representation in the VPM and VPL thalamic nuclei**.

	AP (mm)	L (mm)	D (mm)	*N*
FACE	7.67 ± 0.50	3.18 ± 0.49	8.42 ± 0.81	32
WHISKER	7.69 ± 0.48	3.32 ± 0.44	8.39 ± 0.92	19
FORELIMB	8.28 ± 0.71	4.19 ± 0.50	8.50 ± 0.77	27
HIND LIMB	8.34 ± 0.72	4.68 ± 0.46	8.97 ± 0.61	23
TRUNK	7.42 ± 0.45	5.08 ± 0.42	8.10 ± 0.69	4
NECK	7.92 ± 0.56	3.37 ± 0.55	8.55 ± 0.70	4

Kruskal-Wallis Test followed by Dunn’s Multiple Comparison Test showed statistically significant differences associated with anterior-to-posterior (face vs. forelimb, P < 0.01; face vs. hind limb, P < 0.01; whisker vs. forelimb, P < 0.5; whisker vs. hind limb, P < 0.5) and medial-to-lateral (face vs. forelimb, P < 0.001; face vs. hind limb, P < 0.001; whisker vs. forelimb, P < 0.001; whisker vs. hind limb, P < 0.001; whisker vs. neck, P < 0.001; hind limb vs. trunk, P < 0.05; trunk vs. neck, P < 0.05) but not depth. Mean ± S.E.

Therefore, in each experiment we found a rather consistent topography of the body representation in the somatosensory thalamus. However, when all experiments were combined a large variability was observed. This can be explained by the large cranial variability of ferrets (Lawes and Andrews, 1987). To get a more realistic topography of the somatosensory thalamus we realigned the recording sites (Figures 2–4 bottom, right). In three of nine analyzed experiments we recorded several neurons in the representation of whiskers, forelimb and hindlimb. For an alignment in the frontal plane (Figure 2), we took experiment six (orange symbols) and projected the frontal coordinates of all recording sites on a frontal section of the brain obtained from the same experiment in accordance with stereotaxic values. Then we moved the projection maps obtained in experiment eight (red) and nine (black) to match the center of the whisker representation in the thalamus. After doing that the other body representations appeared to be well aligned with those of experiment six, meaning that the distance between most remote neurons responding to the same type of stimulus was less than 0.5 mm. To complete the alignment we moved the projection maps from the other 6 experiments to match the topography obtained in the reference experiment. The same approach was used to align the topographic representation of the body in the horizontal plane (experiment 8 as reference (red), Figure 3) and in the sagittal plane (experiment 9 as reference (black), Figure 4). To obtain the alignments, the maximal displacement in the lateral dimension was 0.9 mm, in the vertical dimension was 1 mm and in the anterio-posterior dimension it was 1.3 mm.

### Location of first order somatosensory nuclei within ferret thalamus

**Figure 5:**
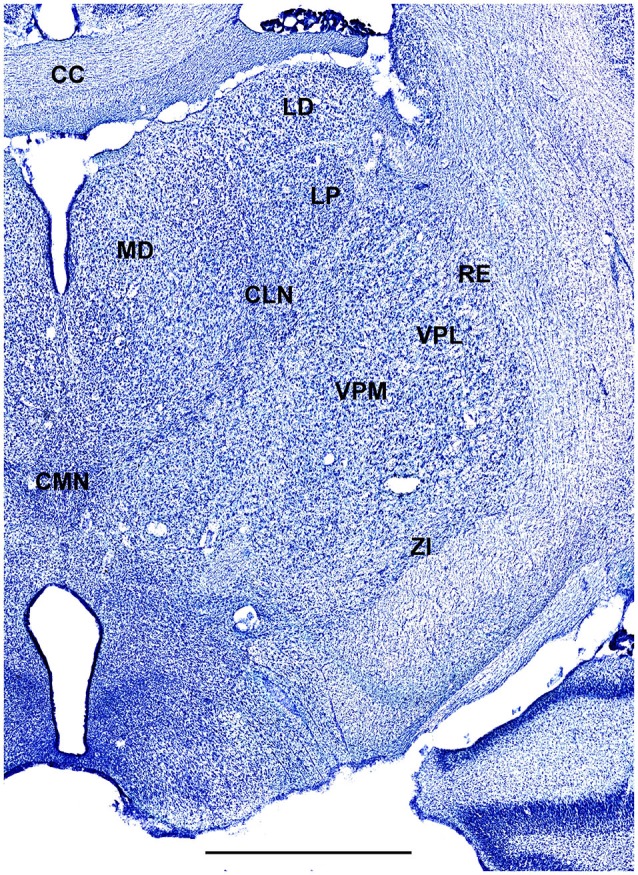
**A fragment of coronal section obtained 9.5 mm anterior from intra aural line showing thalamus**. Scale bar is 2 mm.

**Figure 6:**
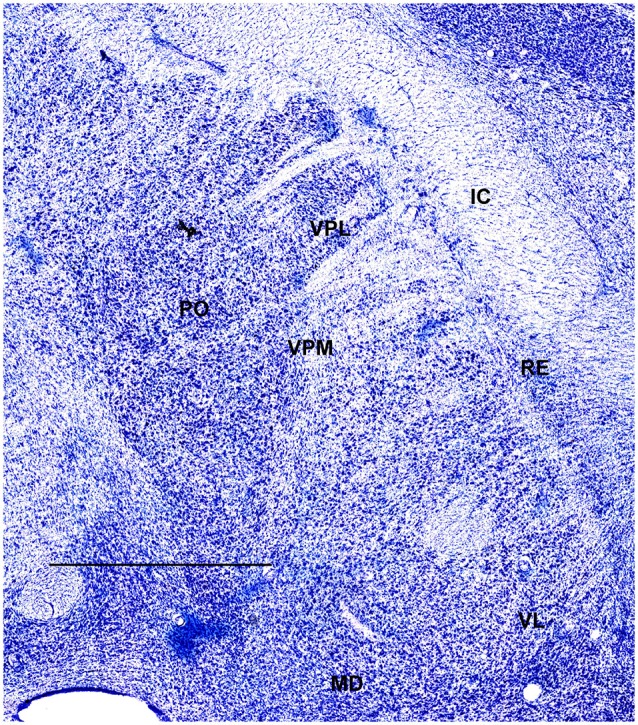
**A fragment of coronal section obtained 7.5 mm dorsal from intra aural line showing thalamus**. Scale bar is 2 mm.

**Figure 7:**
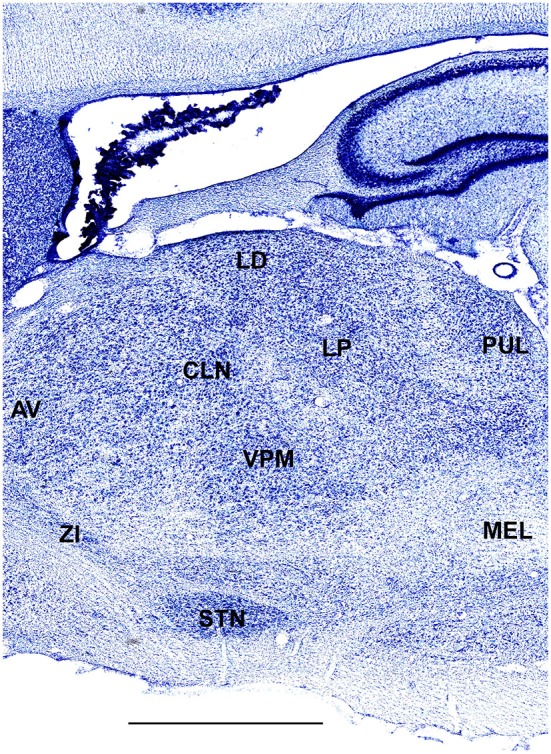
**A fragment of sagittal section obtained 3.6 mm lateral from mid-line showing thalamus**. Scale bar is 2 mm.

In order to define thalamic structures surrounding somatosensory thalamus of ferrets we compared Nissl-stained sections from our experiments with Nissl-stained images from cat brain (Berman and Jones, 1982). We choose cat brain, because like ferrets the cats are carnivores with well-investigated brain morphology and with a size only slightly larger than the size ferrets. In order to enhance visualization of thalamic nuclear subdivision, we took the same images as in Figures 2–4 and digitally increased contrast (Figures 5–7). In coronal view, reticular thalamic nucleus (RE) is seen as a thin line of cells just lateral to VPL nucleus and it outlines the most lateral extent of the thalamus (Figure 5). Medial and superior to somatosensory thalamus are clearly identifiable neuronal groups corresponding to central lateral (CLN) and lateral posterior (LP) nuclei correspondingly. In horizontal view (Figure 6), the RE is seen laterally and anteriorly to somatosensory nuclei. Ventro-lateral (VL) nucleus is located in anterio-medial direction and posterior nucleus is just posterior to VPL. In sagittal view, CLN and LP nuclei are located above VPM and medial lemniscus (MEL) is clearly identifiable ventral and posterior to the VPM nucleus.

## Discussion

We have determined that the thalamic somatosensory representation of the ferret may be divided into VPM and VPL thalamic nuclei based on electrophysiological response evoked by distinct parts of the body surface. The pattern of topographic representation of VPM and VPL nuclei appears to be similar to that described in other large mammals (Rose and Mountcastle, 1952; Loe et al., 1977). There are several features that are specific to ferrets. (a) In the whiskers representation, the neurons responded to stimulation of two whiskers as opposed to rodents in which VPM neurons from each barreloid respond to a different feature, but of one whisker (Timofeeva et al., 2003). (b) As expected, a large proportion of units responded to digits stimulation. (c) We found a clear topological correspondence from forelimb to hindlimb in the medial-to-lateral direction. (d) There was a relatively low level of somatotopic organization for the other body parts (ex: lower jaw, neck). This is not surprising, because previous studies have shown that the responses to the same region of the body can be recorded over large parts of the somatosensory cortex of ferrets (McLaughlin et al., 1998). (e) We demonstrated a large individual variability of the location of the ferret somatosensory thalamus in relation to stereotaxic coordinates; however, the relative spatial relation between different somatosensory thalamic sites was small.

One of the purposes of the study was to obtain some functionally identified location within the thalamus to be used as stereotaxic reference point for the identification of other subcortical structures. Our results suggest that the whisker representation in VPM can be used as an excellent reference point. Another possibility is to use the thalamic representation of forelimbs or hindlimbs. If these locations were used as reference point, the estimated error to identify other thalamic structure in electrophysiological experiments would be in the order of 0.5 mm. If no reference point is used and the electrodes are positioned exclusively based on stereotaxic coordinates that error would be in the order of 2 mm.

## Conflict of interest statement

The authors declare that the research was conducted in the absence of any commercial or financial relationships that could be construed as a potential conflict of interest.
